# The effect of antenatal depression and antidepressant treatment on placental tissue: a protein-validated gene expression study

**DOI:** 10.1186/s12884-019-2586-y

**Published:** 2019-12-05

**Authors:** Åsa Edvinsson, Charlotte Hellgren, Theodora Kunovac Kallak, Helena Åkerud, Alkistis Skalkidou, Elisabet Stener-Victorin, Romina Fornes, Olav Spigset, Susanne Lager, Jocelien Olivier, Inger Sundström-Poromaa

**Affiliations:** 1 Department of Women’s and Children’s Health, Uppsala University, 751 85 Uppsala, Sweden; 2 Department of Immunology, Genetics and Pathology, Uppsala University, 751 85 Uppsala, Sweden; 3 Department of Physiology and Pharmacology, Karolinska Institute, 171 77 Stockholm, Sweden; 4 Department of Clinical Pharmacology, St. Olav University Hospital, 7006 Trondheim, Norway; 5 Department of Clinical and Molecular Medicine, Norwegian University of Science and Technology, 7491 Trondheim, Norway; 6 Neurobiology, Unit Behavioral Neuroscience, Groningen Institute for Evolutionary Life Sciences, University of Groningen, 9747 AG Groningen, The Netherlands

**Keywords:** Antenatal depression, Antidepressant treatment, Selective serotonin reuptake inhibitors, Placental gene expression, TaqMan low-density array, Placental protein expression, Immunohistochemistry

## Abstract

**Background:**

Antenatal depression affects 10–20% of pregnant women. Around 2–4% of European pregnant women use antidepressant treatment, most commonly selective serotonin reuptake inhibitors (SSRIs). Poor pregnancy outcomes, such as preterm birth and low birth weight, have been described in women with antenatal depression and in pregnant women on SSRI treatment. However, the effects of antenatal depression and antidepressant treatment on the placenta are largely unknown. The aim of this work was to compare placental gene and protein expression in healthy women, women with untreated antenatal depression and women on antidepressant treatment during pregnancy.

**Methods:**

Placental samples from 47 controls, 25 depressed and 45 SSRI-treated women were analysed by means of qPCR using custom-designed TaqMan low-density arrays (TLDAs) for 44 genes previously known to be involved in the pathophysiology of depression, and expressed in the placenta. Moreover, placental protein expression was determined by means of immunohistochemistry in 37 healthy controls, 13 women with untreated depression and 21 women on antidepressant treatment. Statistical comparisons between groups were performed by one-way ANOVA or the Kruskal–Wallis test.

**Results:**

Nominally significant findings were noted for *HTR1A* and *NPY2R*, where women with untreated depression displayed higher gene expression than healthy controls (*p* < 0.05), whereas women on antidepressant treatment had similar expression as healthy controls. The protein expression analyses revealed higher expression of HTR1A in placentas from women on antidepressant treatment, than in placentas from healthy controls (*p* < 0.05).

**Conclusion:**

The differentially expressed HTR1A, both at the gene and the protein level that was revealed in this study, suggests the involvement of HTR1A in the effect of antenatal depression on biological mechanisms in the placenta. More research is needed to elucidate the role of depression and antidepressant treatment on the placenta, and, further, the effect on the fetus.

## Introduction

During pregnancy a woman’s body undergoes numerous changes, both physically and mentally. The overall risk of experiencing depressive symptoms during this time period is as common as in the population as a whole, approximately 10–20% [1, 2], whereas major depressive disorder is found in around 4–7% of pregnant women [3–6]. Suffering from depression in pregnancy could have serious consequences for both the mother and the child. Almost 11% of women of childbearing age in Sweden were using antidepressant medication in 2016 [7]. The most commonly prescribed antidepressants in pregnancy are selective serotonin reuptake inhibitors (SSRIs), with a prevalence of around 2–4% in pregnant women in Europe, and 4–10% in North America [8–12]. Poor pregnancy outcomes, such as preterm birth and low birth weight have been reported both in women with antenatal depression and in women who used antidepressant treatment during pregnancy, and it is presently unclear whether it is the SSRI treatment or the depression itself that causes these complications [13–15].

The placenta is a transitory organ that connects the fetus to the mother and acts as a bridge between the maternal and the fetal circulations. The fetal part of the placenta consists of chorionic villous trees that contain a variable amount of fetal vessels and stroma covered by a cytotrophoblast and a syncytiotrophoblast layer. Maternal blood pools into the intervillous space and bathes the villous trees, where feto-maternal exchange takes place [16]. Anchoring villi are attached to the maternal part of the placental tissue, the *decidua basalis*, which is developed from uterine tissue. Placental dysfunction can have devastating consequences for both the mother and the child. Fetal growth can be negatively affected if the placental blood supply is insufficient or if the transport of oxygen and nutrients is affected, which can lead to low birth weight, preterm birth, and birth defects [17–19]. Moreover, the mother is at increased risk of developing conditions such as pre-eclampsia as a result of placental dysfunction, and a history of pre-eclampsia is also associated with abnormalities in the placenta in the current pregnancy [20].

SSRIs have the ability to cross the placenta and have been found in both cord blood and amniotic fluid [21, 22]. Serotonin is involved in embryogenesis [23], placentation [24], and placental vasoconstriction [25], all of which may affect the fetus. In mice, maternal SSRI treatment has been shown to alter multidrug resistance (phosphoglycoprotein) activity both in the placenta and in the fetal and maternal blood-brain barrier, resulting in altered drug transfer into the fetal and maternal brain [26]. Another study in mice revealed a placental serotonin synthetic pathway [27], where serotonin produced in the placenta accumulates in the fetal forebrain during the developmental phase that corresponds to the first and second trimester in humans. This phase includes cortical neurogenesis, migration and initial axon targeting, which are crucial mechanisms for normal neurodevelopment [27].

Regarding the effect of maternal mental illness on placental function, several studies within a review by Gentile and Fusco describe altered gene/protein expression and epigenetic modifications in placenta from depressed mother with or without antidepressant treatment [28]. In addition, studies carried out by our research group have revealed altered gene expression in the fetal part of the placenta in women with depression during pregnancy compared with non-depressed pregnant women [29, 30]. Moreover, use of SSRIs during pregnancy has been found to alter placental gene expression in the fetal part compared with placental gene expression in healthy pregnancy [29, 30]. Oxytocin has an important endocrine role in pregnancy and parturition and methylation of the oxytocin receptor (OXTR) has been linked to mental disorders [31, 32]. Of interest is a study by Galbally et al. suggesting that antidepressant exposure rather than depressive symptoms during pregnancy can alter OXTR methylation in placental tissue [33]. Also, decreased expression of the imprinted gene PEG3 in the placenta have been found associated with maternal depression [34]. Investigators have also described genes involved in the hypothalamic–pituitary–adrenal (HPA) axis that are differentially expressed in placentas from mothers suffering from prenatal stress compared with healthy mothers [35–39]. There are also studies describing associations between maternal depression, the placenta and child behaviour and temperament [40, 41]. However, much remains to be revealed concerning the biological effects of fetal exposure to maternal depression and its treatment. Specifically, more research is needed to elucidate the different effects of antenatal depression and antidepressant treatment on placental function and the offspring. The aim of this work was to compare placental gene and protein expression in healthy women, women with antenatal depression and women on antidepressant treatment during pregnancy. For this purpose, we selected genes that have previously been shown to be involved in mood disorders, and at the same time known to be expressed in the placenta.

## Materials and methods

### Study population

The placental samples for this study were derived from the Biology, Affect, Stress, Imaging, Cognition (BASIC) project, which is a population-based, longitudinal study on psychological well-being during pregnancy and the postpartum period, conducted at the Department of Obstetrics and Gynaecology, Uppsala University Hospital. In the BASIC study all women attending routine ultrasound examination at gestational weeks 16–18 are invited to participate. Exclusion criteria in the BASIC project are (1) inability to communicate adequately in Swedish, (2) protected identity, (3) age less than 18 years, and (4) blood-borne infectious diseases. In Uppsala, all routine ultrasound examinations are performed at Uppsala University Hospital and 97% of pregnant women participate. Moreover, the only delivery ward in the county is at Uppsala University Hospital, thus forming the basis of a population-derived sample. Written informed consent is obtained from women who choose to participate in the BASIC project, and within this consent document women also specify whether or not blood and placental samples may be collected at delivery. Placental tissue was collected between April 2010 and September 2013, and in all, 957 placental samples were collected.

In the BASIC project, women are followed by means of web-based questionnaires at gestational weeks 17 and 32. At both of these times women fill out the Edinburgh Postnatal Depression Scale (EPDS) [42, 43], together with detailed information on previous and ongoing medical treatment, including antidepressant use, smoking, educational level and country of origin. Information on maternal body mass index (BMI) (gestational weeks 10–12), obstetric and perinatal variables is retrieved from the medical records.

The EPDS is a self-administered instrument that contains 10 statements, scored from zero to three, rendering a maximum score of 30. This instrument does not provide a diagnosis of depression as such, but a higher total score increases the likelihood of having a major depressive episode. EPDS statements are based on the past seven days. The sensitivity is relatively low, but the scale has a specificity of 98–99% for major depression at a cut-off score of > 12 points during pregnancy [44]. The internal consistency and test-retest reliability of the EPDS questionnaires had a Cronbach’s alpha of 0.88, and a coefficient of stability of 0.77.

A selection of the women in the BASIC cohort also participated in a psychophysiological sub-study in late pregnancy and early postpartum. As part of this sub-study, women were subjected to a psychiatric interview, the Mini International Neuropsychiatric Interview (MINI), by which a diagnosis of minor or major depressive disorder was established. Blood samples were also drawn at these visits.

For this study, placental samples from 47 healthy controls, 25 women with untreated antenatal depression and 45 women on antidepressant treatment were used. General exclusion criteria for all groups were maternal age > 42 years, alcohol use during pregnancy, any pregnancy complication that would influence, or be a sign of, compromised placental function such as pre-eclampsia, gestational diabetes, pre-pregnancy diabetes, intrauterine growth restriction, offspring born small for gestational age, and gestational age < 35 weeks at delivery.

The definition of being depressed in this study was a diagnosis of depression according to the MINI interview (*n* = 19), or an EPDS score of ≥ 12 in weeks 17 *and* 32, together with a diagnosis of previous major depression according to MINI or according to medical records (*n* = 6). According to the medical records women on antidepressants used their treatment during at least half of the pregnancy in clinically relevant doses. The control subjects had a maximum EPDS score of 11, and no on-going/earlier psychiatric disease according to medical records, and were matched to the depressed and SSRI-treated women with respect to BMI (± 2.0 kg/m^2^) and age (± 5 years).

### Samples

The BASIC placental tissue samples were collected and processed directly after delivery. Two basal-plate biopsy samples from the maternal-fetal interface, of approximately one cm in thickness, were excised from the central part of the placenta containing both the maternal decidua basalis and fetal villous tissue. Calcified areas or infarcts in the tissue were avoided. The tissue samples were carefully rinsed with sterile phosphate-buffered saline (PBS) and put on dry ice within 30 min after delivery. The samples were stored at − 70 °C until further processing. For paraffin-embedded placental samples, tissues were fixed for 24 h in 4% formalin, and stored in 70% ethanol until paraffin-embedding.

A placental sample of 3 mm^3^ was taken from the fetal side of the frozen placental tissue piece for isolation of total RNA using RNeasy mini kits (#74106, Qiagen, Hilden, Germany). The tissue was homogenized and lysed using a rotor-stator homogenizer and RLT-β-mercaptoethanol, according to the manufacturer’s protocol. The amount and quality of RNA were determined by using NanoDrop technology and by Agilent RNA 6000 Nano assays, respectively (Dalcochromtech, Life Technologies Inc., CA, USA).

Single-strand cDNA was synthesized from 250 ng of total RNA using Superscript VILO MasterMix kits (Life Technologies, Paisley, UK), following the manufacturer’s protocol, resulting in a total amount of 250 ng cDNA/sample.

### Low-density Array analysis

Quantitative real-time PCRs were run with custom-designed TaqMan low-density arrays (TLDAs) (Applied Biosystems, Foster city, CA, USA) for 44 genes previously known to be involved in the pathophysiology of depression, and expressed in the placenta (Additional file 1). These genes represent: i) monoaminergic pathways, including genes such as sodium-dependent serotonin transporter (*SLC6A4*), aromatic l-amino acid decarboxylase (*AADC*), and monoamine oxidase A (*MAOA*), all involved in monoamine transport to the fetus [27, 45]; ii) hypothalamic–pituitary–adrenal (HPA) axis function, including genes such as corticotrophin-releasing hormone (*CRH*) and neuropeptide Y (*NPY*), which drive the maternal HPA axis during pregnancy [46], and 11β hydroxysteroid dehydrogenase types 1 and 2 (*HSD11B1* and *2*), responsible for shuttling of cortisol to the fetus [47]; iii) other hormonal systems, including oxytocin (*OXT*) and enzymes involved in progesterone metabolism, such as 5α-reductase types I and II (*SRD5A1* and *2*) and 3α-hydroxysteroid dehydrogenase (*AKR1C4*) [48, 49]; iv) growth factors, including vascular endothelial growth factor (*VEGF*) and brain-derived neurotrophic factor (*BDNF*), and v) genes involved in placental drug metabolism and transport. Finally, we included nerve growth factor (*NGF*) to validate our previous findings [29, 30]. Four genes, *GAPDH*, *TOP1*, *YWHAZ*, and *ABCB1* were included in the arrays as reference genes (Additional file 1).

Each TaqMan LDA consisted of 384 wells and 8 ports (48 wells/assays per port). The 117 samples were loaded to the TLDAs via the ports, one sample per port, which resulted in 15 TLDAs in total. Samples were run as singletons, and the amount of cDNA in each loading port was equivalent to 100 ng of mRNA. The arrays were run according to the manufacturer’s protocol with an ABI Prism 7900HT Sequence Detection System and ABI Prism 7900HT SDS software version 2.4 (Applied Biosystems).

Each assay included a forward primer, a reverse primer, and a TaqMan® MGB probe (Additional file 1) with the reporter FAM™ and the quencher MGB-NFQ. Negative controls consisted of no template (water).

Each placental sample (100 ng cDNA) was diluted with sterile water to a volume of 50 μL, with addition of an equal volume of TaqMan Universal PCR Master Mix (2 ×; Applied Biosystems). The sample was loaded to the TaqMan LDA, which was then centrifuged twice for 1 min at 331×*g.* In cases of excess sample in the fill reservoir the LDAs were spun for an additional 1 min. The final volume in each well after centrifugation was 1.5 μL, which yielded 1.5 ng cDNA per reaction. Real-time RT-PCRs were run with thermal cycling conditions of 2 min at 50 °C, 10 min at 95 °C, followed by 40 cycles of denaturation at 95 °C for 15 s and annealing and extension at 60 °C for 1 min.

### Analysis of real-time RT-PCR data

Manual confirmation of threshold detection was conducted for quality-control purposes. We utilized Ct number as input for our variability analysis among tissue samples for each target. Results for each target in TLDA analysis were quantified concurrently using the same baseline and threshold for a target gene in order to limit inter-plate errors in the analysis. By using NormFinder, GeNorm algorithms and GenEx software (MultiD Analyses) [50], we identified *GAPDH* and *YWHAZ* as the most stable combination of genes to use for normalization in data analysis. Normalization of the data included subtraction of the mean Ct values of the best combination of housekeeping genes from the mean Ct value for each gene in each group (ΔCt). A higher ΔCt value refers to a lower gene expression, and a lower ΔCt value refers to a higher gene expression respectively.

### Immunohistochemistry

Based on availability of paraffin-embedded blocks of placental tissue among women included in the gene-expression analysis, placental-protein expression was determined in 37 healthy controls, 13 women with untreated depression and 21 women on antidepressant treatment. The paraffin-embedded blocks were sectioned (4 μm) and the samples placed on Superfrost slides. The slides were processed according to a standardized immunohistochemistry protocol, with antibody retrieval in 1 × citrate buffer for 10 min in a 650 W microwave oven. An endogenous peroxide-blocking step for 10 min in 3% H_2_O_2_ in ethanol was followed by a non-immune block with 5% normal goat/horse serum in 0.1% bovine serum albumin (BSA) in PBS for one hour at room temperature (RT).

The primary antibodies used in the following step were anti-HTR1A (PA5–28090, rabbit, Thermo Fisher Scientific) and anti-NPY2R (PA1–41576, rabbit, Thermo Fisher Scientific), at dilutions of 1:500 and 1:250, respectively. The antibodies were applied to the slides, which were then kept at 5 °C overnight. As a negative control we used 0.1% BSA in PBS. After the primary antibody step a secondary antibody was applied to the tissue sections for one hour at RT at a dilution of 1:300 in 0.1% BSA in PBS. The secondary antibody used in this study was a biotinylated Goat Anti-Rabbit antibody (Vector labs BA-1000). As a detection method we used a colorimetric system including an enzyme, horseradish peroxidase (HRP) (dilution 1:400, 1 h at RT, Vector labs A-2004), and a substrate, DAB (3,3′-diaminobenzidine) (dilution, chromogen/substrate 1:50, 20 s at RT) (Dako). The enzyme HRP catalyses oxidation of the substrate DAB, resulting in a brown colour in the sample. Mayer’s haematoxylin was applied for staining of the cell nuclei.

### Immunohistochemical scoring

The immunohistochemically stained placental-tissue sections were analysed in terms of staining pattern, distribution, intensity (0–3), and the proportion (%) of stained cells. Two independent scorers carried out the scoring in a blinded manner as regards case/control status. Intensity was defined as negative (0), weak (1), medium (2), or strong (3). The tissue samples were further evaluated by calculating an H-score (histo-score), based on the staining intensity and proportion of stained cells. The H-score takes into account different staining intensities within the same tissue sample, and is assigned using the following formula: [1 × (% of cells with intensity score 1) + 2 × (% of cells with intensity score 2) + 3 × (% of cells with intensity score 3)]. The final score thus ranges from 0 to 300 [51, 52].

### Antidepressant concentration measurement

Maternal blood for the analysis of antidepressant serum concentrations was obtained at the delivery ward prior to the woman’s delivery. Blood samples were centrifuged at 1500 RCF for 10 min and the sera were stored at − 70 °C within 1 h of delivery.

Citalopram, escitalopram, sertraline, and fluoxetine and its active metabolite norfluoxetine were analysed using liquid chromatography–mass spectrometry by methods described previously [53]. In brief, citalopram and escitalopram were extracted from serum by liquid–liquid extraction using a mixture of hexane/butanol/acetonitrile (93/5/2) as extraction solvent. Sertraline, fluoxetine and norfluoxetine were extracted from serum by liquid–liquid extraction using butyl chloride as extraction solvent. All analytes were then quantified on an Agilent MSD 1100 LC-MS system (Agilent Technologies, Palo Alto, CA, USA) after separation on C18 columns, and subsequent detection as pseudomolecular (M + 1) ions. Internal standards were used. Together with the unknown patient samples, each analytical series contained seven calibrators covering therapeutic, subtherapeutic, and toxic concentrations. In addition, six quality-control samples with representative target concentrations were included. The limits of quantification were 5 nmol/L for sertraline and 10 nmol/L for citalopram, escitalopram, fluoxetine and norfluoxetine. Accuracy was controlled routinely with external control samples, and precision was calculated from the quality-control samples. In general, the inter-assay coefficients of variation were less than 10%. The methods were linear in the concentration ranges concerned.

### Statistics

Four genes, *CRHR1*, *NPY*, *SRD5A2* and *AKR1C4* were expressed in less than 50% of the samples, evenly distributed between groups, and were excluded from further analyses. Extreme outliers (> 4.9 SD) were removed from the dataset (*n* = 16 data points).

Demographic data were compared using Chi-square tests, one-way ANOVA, or Kruskal-Wallis test, with Tukey HSD or Dunn’s test as post hoc test, respectively. Testing for normal distribution was done by the Shapiro-Wilk test. While the majority of ΔCt values were normally distributed, some were skewed; thus, data is presented as medians with interquartile ranges (IQRs). Statistical comparisons between groups were performed by one-way ANOVA or the Kruskal–Wallis test, depending on the distribution of the individual variable. When the results of ANOVA were significant, post hoc tests were pursued with the Tukey HSD test for normally distributed variables and the Dunn’s test for skewed variables. In addition, correction for multiple testing was performed according to Bonferroni. Protein expression was compared between groups by the Kruskal–Wallis test, followed by Dunn’s test, and protein expression data is presented as medians (IQR). Correlation between placental gene and protein expression was done by Spearman correlation. Statistical analyses were performed using IBM SPSS software (versions 24.0 and 25.0). Values of *p* < 0.05 were considered statistically significant.

## Results

### Clinical characteristics

Demographic and clinical variables of the study groups are given in Table 1. The three study groups did not differ in terms of age and BMI, but women with untreated depression more often continued smoking during pregnancy, and women on antidepressant treatment more often had previous children in comparison with the healthy controls. In addition, women on antidepressant treatment had a shorter gestational length at delivery than those in the other two study groups.

**Table 1:** Demographic and clinical variables in the study group

Variable	Healthy controls (*n* 47)	Women with untreated depression (*n* 25)	Women on antidepressant treatment (*n* 45)	p*	*p* *untreated depression* vs. *controls*	*p* *antidepressant treatment* vs. *controls*	*p* *antidepressant treatment* vs. *untreated depression*
Age, years	30.3 ± 3.5	30.3 ± 4.9	31.2 ± 4.8	0.560			
BMI, kg/m^2^	25.7 ± 4.7	25.0 ± 4.4	26.7 ± 5.0	0.339			
Nordic origin, n (%)	46 (97.9)	23 (92.0)	42 (93.3)	0.302			
University education, n (%)	34 (82.9)	16 (64.0)	28 (70.0)	0.489			
Parous, n (%)	19 (40.4)	14 (56.0)	30 (66.7)	0.040	0.207	0.012	0.378
Smoking during pregnancy, n (%)	0	3 (12.0)	1 (2.2)	0.024	0.039	0.489	0.127
Mode of delivery				0.460			
Vaginal, n (%)	40 (85.1)	21 (84.0)	34 (75.6)				
Planned CS, n (%)	2 (4.3)	2 (8.0)	7 (15.6)				
Emergency CS, n (%)	5 (10.6)	2 (8.0)	4 (8.9)				
Gestational length, days	282 ± 7	282 ± 8	276 ± 9	0.001	0.995	0.001	0.007
Birthweight, grams	3697 ± 587	3769 ± 427	3640 ± 437	0.571			
EPDS in gestational week 17	2 (1–5)	13 (11–16)	7 (5–11)	0.001	0.001	0.001	0.009
EPDS in gestational week 32	3 (1.5–5)	15 (13–16)	7 (5–12)	0.001	0.001	0.001	0.002

Data presented as mean ± SD, median (IQR) or n (%). Statistics by Chi-square tests*, one-way ANOVA*, or Kruskal-Wallis test*. Post hoc tests by Tukey HSD or Dunn’s test. Frequencies given in relation to available responses. *BMI* Body Mass Index, *CS* Caesarean Section, *EPDS* Edinburgh Postnatal Depression Scale, *SD* standard deviation, *IQR* interquartile range

Women with untreated depression had higher scores of self-rated depression (EPDS) at gestational weeks 17 and 32 in comparison with the controls (Table 1). A similar pattern was noted in women on antidepressant treatment, although their self-rated depression scores at the same time points were lower than among the untreated depressed women (*p* = 0.009 and *p* = 0.002, respectively).

The 45 women on antidepressant treatment were most often using sertraline (*n* = 17), followed by fluoxetine (*n* = 13), citalopram (n = 13) and escitalopram (*n* = 2). According to the medical records, 39 (86.7%) had been using antidepressant treatment throughout pregnancy, two women during the first half of pregnancy and four women during the second half of pregnancy. Blood samples at parturition were available in 41 cases. Of these, 28 showed detectable concentrations of antidepressant drugs (Table 2).

**Table 2:** Maternal serum concentrations of antidepressant drugs at parturition in 41 women from whom blood samples were available

Antidepressant drug	Analyzed, n	Detectable concentration, n	Mean ± SD nmol/l	min – max nmol/l
Sertraline	15	8	41.1 ± 71.0	8–216
Fluoxetine^a^	12	11	420 ± 242	36–739
Citalopram	12	9	102 ± 55	21–208
Escitalopram	2	0	–	–

^a^Including its active metabolite norfluoxetine

### Placental gene expression

Overall, following correction for multiple testing, none of the examined genes were differentially expressed between healthy controls, women with untreated depression and women on antidepressant treatment (Table 3).

**Table 3:** Gene expression in placental tissue from healthy controls, women with untreated depression and women on antidepressant treatment

	Healthy controls (*n* 47)		Women with untreated depression (*n* 25)		Women on antidepressant treatment (*n* 45)			
Gene	n	Median (IQR)	n	Median (IQR)	n	Median (IQR)	*p*	*p*_bonferroni_
*NPY2R*	40	12.3 (10.1–13.3)	20	9.7 (9.1–11.7)	37	10.9 (9.3–12.6)	0.024	0.960
*NGF*	47	8.8 (8.1–9.5)	25	8.4 (8.3–8.9)	45	8.3 (7.8–9.0)	0.049	1.984
*HTR1A*	34	10.8 (9.6–12.3)	21	9.0 (7.7–11.3)	27	10.4 (8.8–11.7)	0.049	1.988
*OXTR*	47	9.5 (8.4–11.1)	25	9.3 (7.4–10.0)	45	8.9 (7.9–10.3)	0.081	
*BDNF*	47	6.3 (5.9–6.8)	25	6.0 (5.2–6.4)	45	6.4 (5.8–6.7)	0.091	
*CRH*	47	0.7 (−0.2–2.3)	24	0.1 (−1.0–1.1)	45	1.0 (− 0.1–2.1)	0.102	
*NR3C1*	47	3.0 (2.6–3.4)	25	2.8 (2.0–3.0)	45	2.8 (2.3–3.2)	0.133	
*MAOA*	47	1.0 (0.7–1.6)	25	0.7 (0.3–1.1)	45	1.1 (0.6–1.5)	0.153	
*TPH1*	46	11.7 (10.7–12.7)	24	12.2 (11.3–12.7)	44	11.6 (10.8–12.3)	0.189	
*RAF-1*	47	5.1 (4.7–5.4)	25	4.8 (4.5–5.2)	45	5.0 (4.7–5.3)	0.193	
*HTR3E*	47	9.9 (8.9–10.8)	24	9.1 (8.7–10.4)	45	9.9 (8.7–10.8)	0.195	
*ROCK2*	47	3.0 (2.7–3.3)	25	2.9 (2.5–3.1)	44	2.9 (2.5–3.3)	0.204	
*AADC*	33	11.4 (10.0–12.3)	19	11.5 (8.8–13.9)	30	10.8 (8.8–11.9)	0.206	
*OXT*	32	12.9 (11.4–14.0)	18	12.4 (11.1–12.7)	25	12.6 (11.5–13.2)	0.280	
*SRD5A1*	47	9.2 (8.8–9.6)	25	9.0 (8.6–9.4)	45	9.2 (8.7–9.6)	0.301	
*ROCK1*	46	4.1 (3.8–4.4)	25	3.8 (3.7–4.2)	45	3.8 (3.5–4.1)	0.313	
*COMT*	45	2.9 (2.2–3.2)	25	3.1 (2.8–3.3)	43	2.9 (2.5–3.4)	0.318	
*TPH2*	46	10.4 (9.3–12.2)	25	11.1 (10.4–11.7)	44	11.3 (10.2–12.1)	0.356	
*HSD11B1*	47	5.6 (4.8–6.4)	25	5.8 (4.2–6.3)	45	5.9 (5.2–6.4)	0.426	
*CYP3A4*	26	12.0 (9.5–13.5)	14	11.3 (9.9–12.7)	19	11.1 (8.6–12.5)	0.456	
*CYP2D6*	47	8.7 (7.9–9.7)	25	8.7 (7.6–9.1)	45	8.8 (8.0–9.4)	0.478	
*GABRG2*	43	9.9 (8.9–10.7)	23	10.3 (9.0–11.8)	42	9.7 (8.7–11.3)	0.478	
*CYP1A2*	25	11.7 (10.7–14.1)	19	10.9 (9.8–12.1)	29	11.4 (9.6–13.1)	0.506	
*PTGDS*	47	2.0 (1.5–2.7)	25	1.9 (1.1–2.6)	45	1.9 (1.0–2.6)	0.539	
*HSD11B2*	47	2.5 (1.7–3.3)	24	1.9 (1.6–2.6)	45	2.0 (1.4–3.1)	0.573	
*SLC6A2*	47	3.6 (3.1–4.4)	25	3.5 (3.1–4.2)	45	3.7 (3.5–4.3)	0.624	
*SLC6A4*	46	3.7 (3.3–4.1)	24	3.8 (3.5–4.1)	45	3.8 (3.4–4.4)	0.669	
*CDH2*	47	10.1 (8.3–11.8)	25	10.0 (8.8–11.8)	44	9.4 (8.5–11.0)	0.685	
*NTRK1*	37	10.1 (7.5–11.8)	18	9.0 (7.3–11.4)	31	9.5 (8.0–10.7)	0.724	
*ALAD*	47	4.6 (4.4–4.9)	25	4.6 (4.3–4.9)	45	4.6 (4.3–5.0)	0.726	
*NPY1R*	47	9.6 (9.0–10.0)	25	9.6 (8.7–10.0)	45	9.6 (8.5–10.5)	0.769	
*CREB1*	47	4.2 (3.7–4.4)	25	3.9 (3.7–4.4)	45	4.0 (3.7–4.4)	0.824	
*CRHBP*	47	6.5 (5.4–7.6)	25	6.4 (5.3–8.0)	45	6.7 (5.5–8.1)	0.851	
*ABCB1*	47	5.3 (4.1–5.8)	25	4.8 (4.1–5.9)	45	5.1 (4.1–6.7)	0.855	
*VEGFA*	47	4.6 (3.6–5.4)	25	4.5 (3.6–5.4)	45	4.4 (3.6–5.0)	0.860	
*HTR7*	46	10.1 (9.1–11.6)	25	10.2 (9.2–10.9)	45	10.1 (8.9–10.8)	0.879	
*PGF*	47	0.1 (−1.0–0.7)	25	−0.1 (−1.0–0.9)	45	−0.1 (− 0.6–0.3)	0.893	
*CCK*	46	10.4 (8.4–11.3)	24	10.2 (8.2–11.3)	45	9.4 (8.1–11.4)	0.923	
*NTRK2*	47	9.2 (8.2–9.9)	24	9.1 (8.6–9.9)	44	9.0 (8.4–9.8)	0.956	
*VIP*	46	10.1 (9.2–10.9)	25	9.8 (9.4–10.5)	45	9.9 (9.4–10.6)	0.971	

Data presented as median ΔCt (IQR). Statistical tests by one-way ANOVA or Kruskal Wallis test, depending on normal distribution. Genes sorted according to the degree of statistical significance

**Fig. 1:**
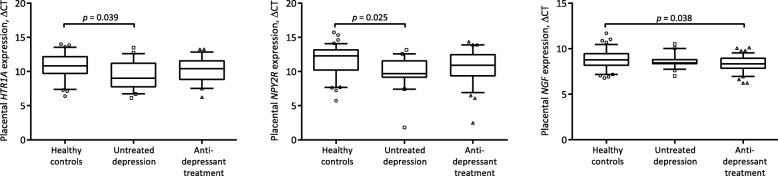
Placental gene expression (median ΔCt, IQR) in women with antenatal depression (*n* = 25), antidepressant treatment (*n* = 45), and healthy controls (*n* = 47). Women with untreated depression had higher gene expression of *HTR1A*, *p* = 0.039 (Tukey HSD), and *NPY2R*, *p* = 0.025 (Dunn’s test), than healthy controls. The gene expression of *NGF* was higher in women with antidepressant treatment than in healthy controls, *p* = 0.038 (Tukey HSD)

Nominally significant findings, i.e. not corrected for multiple comparisons, were noted for *HTR1A* (5-hydroxytryptamine (5-HT)/serotonin receptor 1A), *NGF* and *NPY2R* (neuropeptide Y2 receptor) (Table 3). These findings remained for *HTR1A* and *NPY2R* when analyses were restricted to women with detectable serum concentrations of antidepressant drugs at delivery (median ΔCt for *HTR1A* of 9.1 (IQR 7.9–11.8), *p* = 0.043 and median ΔCt for *NPY2R* of 10.6 (IQR 8.4–12.4), *p* = 0.019). Post hoc analyses revealed that women with untreated depression had higher gene expression of *HTR1A*, (*p* = 0.039) and *NPY2R* (*p* = 0.025) than healthy controls, whereas women on antidepressant treatment had similar expression levels of *HTR1A* and *NPY2R* as healthy controls (Fig. 1). No differences in serotonin (*SLC6A4*) or noradrenaline (*SLC6A2*) transporters or in any of the enzymes associated with serotonin synthesis or degradation (tryptophan hydroxylase 1 (*TPH1*), and 2 (*TPH2*), *AADC*, catechol-O-methyltransferase (*COMT*) and *MAOA*)) were noted between the groups. Further, post hoc analyses revealed that placental gene expression of *NGF* was higher in women with antidepressant treatment than in healthy controls (*p* = 0.038; Fig. 1). The placental NGF gene expression was higher, but not statistically significant, when restricting the analysis to women with detectable levels of antidepressant drugs at delivery (median ΔCt for *NGF* of 8.3 (IQR 7.8–8.9), *p* = 0.056).

Further analyses were conducted within the group of women on antidepressant treatment, comparing those with and without detectable serum concentrations. With the exception of *CYP3A4*, which showed greater expression in women with detectable serum concentrations of antidepressants (ΔCt 10.5 (IQR 7.6–11.7) vs. ΔCt 12.5 (IQR 11.1–13.1), *p* = 0.010), no differentially expressed placental genes were detected between these two groups (data not shown).

### Placental protein expression

**Fig. 2:**
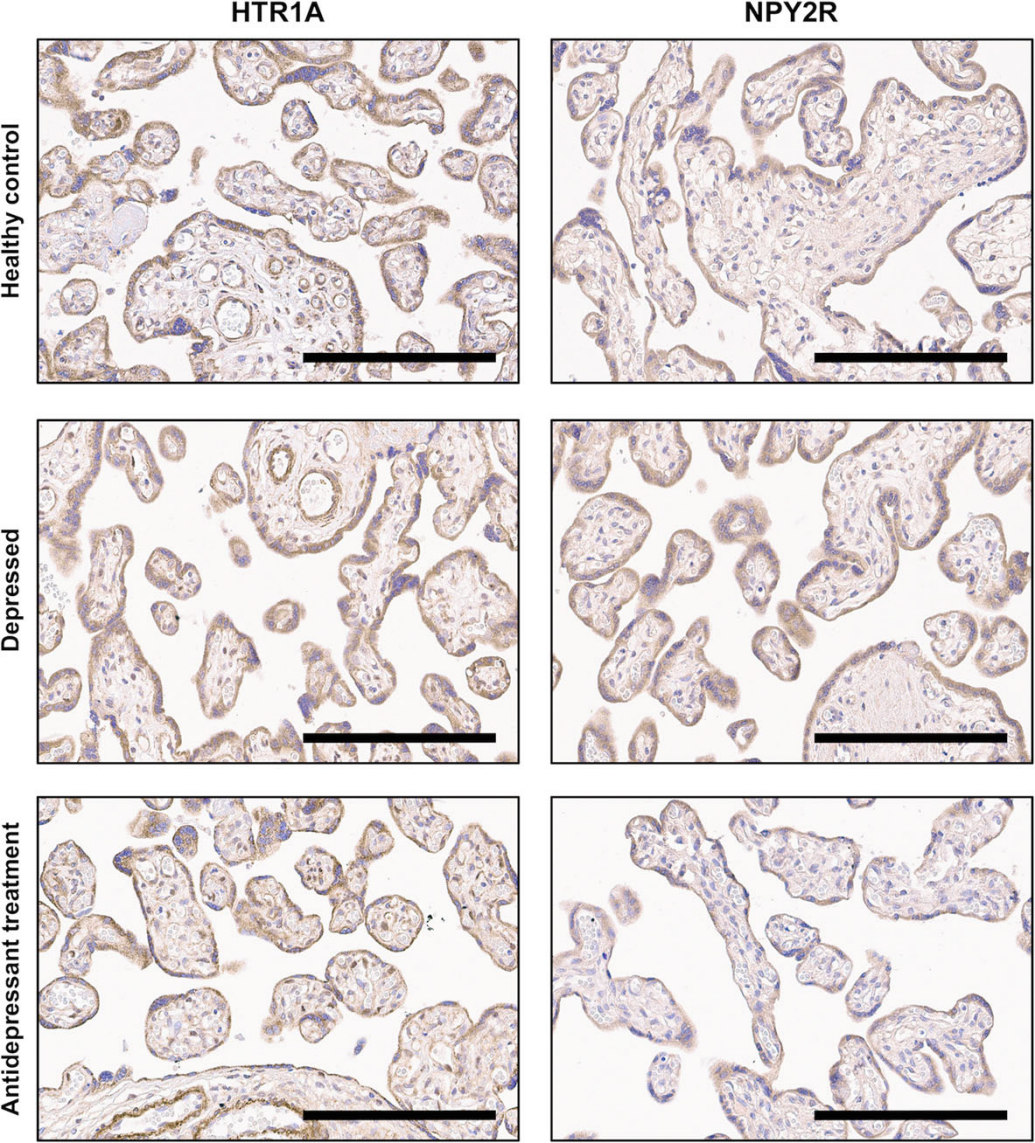
Immunohistochemically stained placental tissue from representative women with antenatal depression, antidepressant treatment, and healthy controls. Antibodies: anti-HTR1A (PA5–28090, rabbit, Thermo Fisher Scientific, dilution 1:500), anti-NPY2R (PA1–41576, rabbit, Thermo Fisher Scientific, dilution 1:250). Scale bar = 200 μm. Objective 20x

**Fig. 3:**
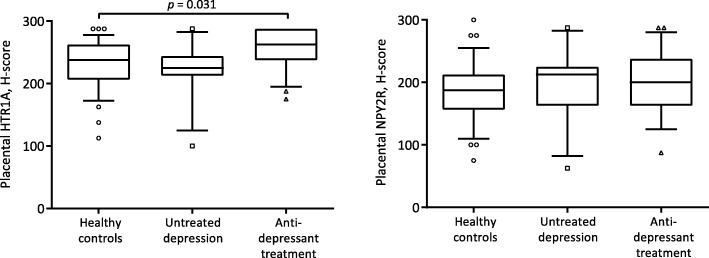
Protein expression (median H-score, IQR) in placental trophoblasts in women with antenatal depression (*n* = 13), antidepressant treatment (*n* = 21), and healthy controls (*n* = 37). The highest HTR1A expression was noted in women on antidepressant treatment, in comparison with healthy controls, *p* = 0.031 (Dunn’s test). No difference between groups in placental NPY2R protein expression was confirmed

Genes nominally significantly different between groups were selected for placental protein expression determination. NGF was not included in the protein analyses, as this has previously been reported by our group [29]. Immunohistochemical staining of HTR1A showed medium-strong intensity in placental trophoblasts and endothelial cells, and medium intensity in stromal cells, whereas staining of NPY2R displayed medium intensity in trophoblasts and weak intensity in endothelial and stromal cells (Fig. 2). HTR1A staining intensity in trophoblasts differed between groups (Table 4), and the difference remained significant in analyses restricted to the treated women with detectable antidepressant serum levels, compared with healthy controls and untreated depressed women (*p* = 0.043). The highest level of HTR1A expression was noted in women on antidepressant treatment, in comparison with healthy controls (*p* = 0.031; Fig. 3). No difference in endothelial and stromal-cell expression of HTR1A was noted between groups. No correlation between placental gene and protein expression of HTR1A was noted (trophoblasts, rho = 0.004, *p* = 0.98; endothelial cells, rho = − 0.18, *p* = 0.27; stromal cells, rho = − 0.06, *p* = 0.71).

**Table 4:** Placental protein expression of HTR1A and NPY2R in healthy controls, women with untreated depression and women on antidepressant treatment

	Healthy controls (*n* 37)	Women with untreated depression (*n* 13)	Women on antidepressant treatment (*n* 21)	*p*
HTR1A				
Trophoblasts	238 (206–263)	225 (213–244)	263 (238–288)	0.017
Endothelial cells	225 (175–238)	225 (169–263)	225 (206–263)	0.284
Stroma cells	175 (138–213)	175 (156–194)	175 (175–231)	0.258
NPY2R				
Trophoblasts	188 (156–213)	213 (163–225)	200 (163–238)	0.743
Endothelial cells	63 (50–63)	63 (50–113)	50 (50–81)	0.351
Stroma cells	50 (50–63)	63 (50–113)	50 (50–81)	0.370

Data presented as median H-score (IQR). Statistics by Kruskal-Wallis test

No difference between groups in placental NPY2R protein expression was confirmed. Gene expression of NPY2R was negatively correlated with corresponding protein expression in placental endothelial and stromal cells (trophoblasts, rho = − 0.11, *p* = 0.43; endothelial cells, rho = − 0.35, *p* = 0.009; stromal cells, rho = − 0.30, *p* = 0.025).

## Discussion

Following correction for multiple testing, our study did not reveal any significant differences in placental gene expression, of the 44 tested genes, between women with antenatal depression, women with antidepressant treatment, and healthy controls. However, given the pregnancy outcomes at stake, i.e. placental function and consequent fetal well-being, it can be argued that a statistically correct approach may conceal clinically relevant findings. For this reason, we have also reported on the few nominally significant transcripts that were detected primarily in women with antenatal depression. All in all, the enhanced gene expression of *HTR1A* in placentas from women with untreated depression, together with the stronger immunohistochemical staining of HTR1A in the treatment group, could strengthen the theory of the involvement of *HTR1A* in maternal depression and placental function. *HTR1A* has previously been identified in placental tissue [54]. Serotonin receptors in the placenta are important for maintenance of the normal development of the fetal brain [55, 56]. Alterations in the placental expression of serotonin receptors have been associated with maternal stress [56] and infant neurodevelopment [57]. While *HTR1A* has previously been described in the trophoblasts of human placenta, at present no information on the role of this receptor in placental function has been published. Notably, the observed differences in placental HTR1A gene and protein expression were not accompanied by changes in the serotonin or noradrenaline transporters or in any of the enzymes associated with serotonin synthesis or degradation in our study cohort, at least at gene-expression level.

5-HT_1A_ receptors can be found as both heteroreceptors and autoreceptors [58–60]. Upon binding of the neurotransmitter to the autoreceptor, a negative feedback loop inhibits further release of the neurotransmitter [61]. Prolonged administration of SSRIs will decrease the autoreceptors’ sensitivity for neurotransmitters, leading to an elevated concentration of synaptic serotonin [61, 62]. While presynaptic 5-HT_1A_ autoreceptors may delay the effect of antidepressant treatment, the stimulation of postsynaptic 5-HT_1A_ receptors in corticolimbic networks is beneficial for antidepressant action [63]. Moreover, polymorphisms in the gene encoding 5-HT_1A_ [64, 65], and altered regulation of 5-HT_1A_ [66] have been associated with depression. A study by Olivier et al. has revealed increased 5-HT_1A_ receptor sensitivity in prenatally fluoxetine-exposed rats, which may, in part, explain the neurodevelopmental changes observed in these animals [67].

In this study, *NGF* gene expression was higher in placental tissue from women using antidepressants during pregnancy. This finding is in line with previous data, where we showed that NGF protein expression was increased in both trophoblasts and endothelial cells from women using antidepressants during pregnancy compared with NGF protein expression in placentas from depressed and healthy controls [29]. Together, these results indicate that SSRI use during pregnancy may affect NGF signalling in the placenta.

The neuropeptide Y2 receptor *(NPY2R)*, which exhibited enhanced gene expression in untreated depressed women, is known to have appetite-inhibiting properties [68, 69]. In addition to food intake, members of the NPY family exert effects on psychomotor activity, regulation of endocrine secretion, energy homeostasis, and effects on the cardiovascular system [68]. There are also studies showing increased levels of NPY in pre-eclamptic women, suggesting involvement in placental function [70]. NPY has been identified in the brain and placenta in rats [71]. Members of the NPY family have also been implicated in CNS functions in primates, where, for example, activation of NPY2R is thought to exert anxiogenic actions [72]. Our results, however, revealed unchanged protein expression in the placenta in relation to the exposures involved. Thus, this finding needs replication before any meaningful interpretations can be made.

The major strengths of this study include the sample of placentas from obstetrically healthy women with stringent definition of antenatal depression, and women on antidepressant treatment. Further, the use of SSRIs is validated by serum concentrations of the actual drug in question. Additional strengths are age- and BMI-matched controls, and validation of the gene-expression results at a protein level by immunohistochemistry. Even so, some limitations need to be mentioned. The use of human placentas inevitably introduces great variability, in this study seen in the various types, doses and duration of antidepressant treatment. Similarly, the severity and duration of antenatal depression also differed between women. This heterogeneity may influence and potentially dilute important findings, but on the other hand, restriction to more stringent samples would affect power in individual analyses. Another limitation is the lower number of samples available for protein analysis compared with those available for gene expression analysis (71 out of 117). Only 28 of 41 women had detectable serum concentrations of an antidepressant drug at delivery. While the serum concentrations validate the medical reports of ongoing SSRI use, it should be stressed that non-confirmation of SSRI treatment in serum does not necessarily mean the placenta has not been exposed to treatment during pregnancy. Many women are aware of neonatal complications from SSRI use during late pregnancy [73], and some discontinue in preparation for delivery to avoid such problems [74, 75]. Clearly, no difference in results was noted when analyses were restricted to women with confirmed SSRI use, and there were also no differences in placental gene expression between women with detectable and non-detectable antidepressant serum concentrations.

Investigators have described associations between poor mental health, impaired placental function, and poor pregnancy outcomes such as preterm birth and low birth weight [13–15], but the underlying biological mechanisms are not fully understood. More research is required to elucidate the causal relationships between these factors. Our findings may be interpreted as being both reassuring and worrying for women who suffer from antenatal depression and need treatment for their condition. The genes investigated were primarily chosen because they have been linked to depression in various human and animal studies. Thus, at the gene level, it may not be surprising that the findings of interest were noted only among women with antenatal depression, whereas SSRI users had similar gene-expression levels as the healthy controls. While this finding may appear reassuring, potentially suggesting that treatment of antenatal depression normalizes placental gene expression, it is contradicted by the findings at the protein level. If we had chosen genes on the basis of their association with antidepressant use, we might have revealed a different picture. Further reassuring findings were that some of the genes we studied are known to have an impact on long-term fetal health, such as HSD11B1 and 2, which control the shuttling of cortisol to the fetus. While maternal stress is known to affect this placental enzyme [35–37, 39], we found no evidence that antenatal depression or antidepressant treatment had such effects. Notably, however, gene expression does not equal function, and previous studies from our group have demonstrated that the maternal cortisone:cortisol ratio, as a functional measure of this enzyme, is positively associated with birth weight in women with psychiatric disease [76]. Of concern, this study has demonstrated differential expression of serotonin receptor 1A and NPY2 receptor, both of which currently are of unclear relevance as regards placental function. Previous microarray studies have demonstrated a multitude of genes that are differentially expressed, especially in women on antidepressant treatment [30], and absence of findings in our study should not be interpreted as the full picture, or that SSRI use during pregnancy is without harm. Clearly, further studies in this area are needed, and the review by Gentile and Fusco describes the importance in more rigorous assessments including ultrasound studies on fetal responses to maternal mood and antidepressant medication and also analyses of epigenetic modifications associated with antenatal depression and antidepressant treatment [28].

## Conclusion

The differential expression of HTR1A at both the gene and protein level that was revealed in this study suggests the involvement of HTR1A in the effect of antenatal depression on biological mechanisms in the placenta. More research is needed to elucidate the roles of depression and antidepressant treatment on placental gene and protein expression, especially as regards HTR1A, and, further, the effect on the fetus.

## Supplementary information

**Additional file 1: Table S1.** Gene symbols, gene names, TaqMan probes.

## Data Availability

The data generated in this study can be found in the Figshare repository https://figshare.com/s/c0d6bdb9dd00d777ff8e.

## Abbreviations

AADC: aromatic l-amino acid decarboxylase
AKR1C4: 3α-hydroxysteroid dehydrogenase
BASIC: Biology, Affect, Stress, Imaging, Cognition
BDNF: brain-derived neurotrophic factor
BMI: body mass index
BSA: bovine serum albumin
COMT: catechol-O-methyltransferase
CRH: corticotrophin-releasing hormone
DAB: 3,3′-diaminobenzidine
EPDS: Edinburgh Postnatal Depression Scale
HPA: hypothalamic–pituitary–adrenal
HRP: horseradish peroxidase
HSD11B: 11 hydroxysteroid dehydrogenase
HTR1A: 5-hydroxytryptamine (5-HT)/serotonin receptor 1A
IQR: interquartile range
MAOA: monoamine oxidase A
MINI: Mini International Neuropsychiatric Interview
NGF: nerve growth factor
NPY2R: neuropeptide Y2 receptor
OXTR: oxytocin receptor
PBS: phosphate-buffered saline
RT: room temperature
SLC6A2: sodium-dependent noradrenaline transporter
SLC6A4: sodium-dependent serotonin transporter
SRD5A: 5α-reductase
SSRI: selective serotonin reuptake inhibitor
TLDA: TaqMan low-density array
TPH: tryptophan hydroxylase
VEGF: vascular endothelial growth factor
