# Supplementary material for: The effect of antenatal depression and antidepressant treatment on placental tissue: a protein-validated gene expression study
Source: BMC Pregnancy Childbirth. 2019 Dec 5;19:479. doi: 10.1186/s12884-019-2586-y (PMC6896358; doi:10.1186/s12884-019-2586-y)
Supplement: Supplementary file 1 — Additional file 1: Table S1. Gene symbols, gene names, TaqMan probes. [file 12884_2019_2586_MOESM1_ESM.docx]

Supplementary table 1. Gene symbols, gene names, TaqMan probes.

|  | Gene symbol | Gene name | TaqMan-id |
| --- | --- | --- | --- |
| Monoamines | *SLC6A4* | serotonin transporter | Hs00984349_m1 |
|  | *HTR1A* | serotonin receptor 1A (5-HT1A) | Hs00265014_s1 |
|  | *HTR3E* | serotonin receptor 3 (5-HT3) | Hs00704511_s1 |
|  | *HTR7* | serotonin receptor 7 (5-HT7) | Hs00909028_g1 |
|  | *SLC6A2* | norepinephrine transporter | Hs00426573_m1 |
|  | *COMT* | catechol-O-methyltransferase | Hs00241349_m1 |
|  | *AADC* | aromatic l-amino acid decarboxylase/  dopa decarboxylase | Hs01105048_m1 |
|  | *TPH1* | tryptophan hydroxylase 1 | Hs00188220_m1 |
|  | *TPH2* | tryptophan hydroxylase 2 | Hs00998777_m1 |
|  | *MAOA* | monoamine oxidase A | Hs00165140_m1 |
| HPA axis | *CRH* | corticotropin releasing hormone | Hs01921237_s1 |
|  | *CRHBP* | corticotropin releasing hormone binding protein | Hs00181810_m1 |
|  | *CRHR1* | corticotropin releasing hormone receptor 1 | Hs00366363_m1 |
|  | *NPY* | neuropeptide Y | Hs00173470_m1 |
|  | *NPY1R* | neuropeptide Y receptor Y1 | Hs00702150_s1 |
|  | *NPY2R* | neuropeptide Y receptor Y2 | Hs01921296_s1 |
|  | *HSD11B1* | 11beta hydroxysteroid dehydrogenase type 1 | Hs01547870_m1 |
|  | *HSD11B2* | 11beta hydroxysteroid dehydrogenase type 2 | Hs00388669_m1 |
|  | *NR3C1* | nuclear receptor subfamily 3, group C, member 1 (glucocorticoid receptor) | Hs00353740_m1 |
| GABA and neurosteroids | *GABRG2* | GABAA receptor γ2 subunit | Hs00168093_m1 |
| Other hormones,  and rate limiting enzymes | *OXT* | oxytocin/neurophysin I prepropeptide | Hs00792417_g1 |
|  | *OXTR* | oxytocin receptor | Hs00168573_m1 |
|  | *SRD5A1* | 5α-reductase type I | Hs00602694_mH |
|  | *SRD5A2* | 5α-reductase type II | Hs00165843_m1 |
|  | *AKR1C4* | 3α-hydroxysteroid dehydrogenase | Hs00559542_m1 |
|  | *VIP* | vasoactive intestinal peptide | Hs00175021_m1 |
|  | *CCK* | cholecystokinin | Hs00174937_m1 |
| Growth factors | *VEGFA* | vascular endothelial growth factor | Hs00900055_m1 |
|  | *BDNF* | brain derived neurotrophic factor | Hs02718934_s1 |
|  | *NTRK2* | BDNF receptor (TrkB) | Hs00178811_m1 |
|  | *PGF* | Placenta growth factor | Hs00182176_m1 |
|  | *NGF* | Nerve growth factor | Hs00171458_m1 |
|  | *ROCK1* | Rho-associated, coiled-coil containing protein kinase 1 | Hs01127699_m1 |
|  | *ROCK2* | Rho-associated, coiled-coil containing protein kinase 2 | Hs00178154_m1 |
|  | *NTRK1* | NGF receptor (Trk-A) | Hs01021011_m1 |
|  | *RAF1* | Raf-1 | Hs00234119_m1 |
|  | *CREB1* | cAMP responsive element binding protein 1 | Hs00231713_m1 |
| Placental drug transport, and metabolism | *CYP2D6* | cytochrome P450, family 2, subfamily D, polypeptide 6 | Hs02576168_g1 |
|  | *CYP3A4* | cytochrome P450, family 3, subfamily A, polypeptide 4 | Hs00604506_m1 |
|  | *CYP1A2* | cytochrome P450, family 1, subfamily A, polypeptide 2 | Hs00167927_m1 |
|  | *ABCB1* | encoding for P-glycoprotein | Hs00184500_m1 |
| Anxiety/stress | *CDH2* | cadherin 2, type 1, N-cadherin (neuronal) | Hs00983056_m1 |
|  | *ALAD* | aminolevulinate, delta-, dehydratase | Hs00163592_m1 |
|  | *PTGDS* | prostaglandin D2 synthase 21kDa (brain) | Hs00168748_m1 |
| Reference genes | *GAPDH* | glyceraldehyde-3-phosphate dehydrogenase | Hs99999905_m1 |
|  | *TOP1* | topoisomerase DNA 1 | Hs00243257_m1 |
|  | *YWHAZ* | tyrosine 3-monooxygenase/tryptophan 5-monooxygenase activation protein, zeta polypeptide | Hs03044281_g1 |
|  | *ACTB* | actin, beta | Hs01060665_g1 |
